# Outbreak of Salmonellosis Associated with Consumption of Pulled Pork at a Church Festival — Hamilton County, Ohio, 2010

**Published:** 2014-01-03

On June 18, 2010, Hamilton County Public Health (HCPH), a local health department in Ohio, began receiving reports of gastrointestinal illness from persons who attended a church festival held during June 11–13 in a suburban community of Hamilton County. HCPH investigated and confirmed the existence of a foodborne outbreak associated with consumption of pulled pork prepared in a private home and sold at the church festival. Sixty-four attendees with gastroenteritis were identified. *Salmonella enterica* serotype Typhimurium (*Salmonella* Typhimurium) was found in stool specimens from three patients; no other pathogen was found. Because the outbreak was identified after the church festival had concluded, the environmental investigation was limited to interviews of food handlers. The primary public health interventions consisted of 1) active surveillance for additional cases of salmonellosis associated with the festival, 2) consultation with the festival organizers and food vendors to ensure the pork product was not resold or consumed elsewhere, 3) education of the festival organizers and food vendors about relevant public health regulations and food safety practices, 4) traceback of the implicated product to the retailer in Indiana, and 5) notification of the Indiana State Department of Health. The results of the investigation call attention to the public health implications of unregulated food service at events such as church festivals, which generally are exempt from public health inspection and licensure in Ohio. Food sold in such environments might place populations at risk for foodborne illness.

During June 11–13, 2010, an estimated 9,000 persons attended a church festival held in a suburban community of Hamilton County, Ohio. Fifteen vendors sold food at the church festival; none were licensed or inspected by HCPH. In Ohio, religious organizations generally are exempt from standard licensure requirements (Ohio Revised Code) (1).

On June 14, symptoms of gastrointestinal illness were noted by the index patient who attended the church festival and who identified two household contacts who also were ill. On June 18, HCPH began an investigation of an outbreak of gastrointestinal illness associated with the church festival and reported the outbreak to the Ohio Department of Health. The purpose of the investigation was to determine the magnitude, cause, and source of the outbreak. Data from initial interviews of ill persons who also attended the church festival indicated that the route of transmission was likely foodborne. The outbreak case definition was a gastrointestinal illness (i.e., vomiting and/or diarrhea) with onset during June 13–18 in a person who had attended the church festival.

Cases were sought with the assistance of the church festival coordinator, who provided information on 22 attendees with gastrointestinal illness who had complained directly to the church. On June 21, a total of 64 persons whose illnesses met the outbreak case definition were interviewed using a standard hypothesis-generating questionnaire. Based on these interviews, typical symptoms were vomiting and diarrhea with onset within 24 hours of the festival closing (June 13) and a duration >24 hours. Several ill persons reported that they believed their illness resulted from consuming pulled pork, coleslaw, or both, served by a single vendor on the last day of the festival. When interviewed, the vendor reported selling 123 servings of pulled pork, some with coleslaw.

*Salmonella* was confirmed as the outbreak pathogen on June 24, when *Salmonella* was cultured from the stool specimens of three patients whose illnesses met the outbreak case definition. Two of the five patients who had stool specimens cultured were negative for bacterial pathogens, including *Salmonella*, *Shigella*, Shiga toxin–producing *Escherichia coli*, and *Campylobacter*. Although the two patients had negative stool cultures, they met the outbreak case definition and reported that they had begun treatment with trimethoprim-sulfmethoxazole before producing a stool specimen.

A matched case-control study was conducted to ascertain the primary risk factor or factors associated with the gastrointestinal illness. Most of the case-patients were identified after being referred by church staff members or a person who had been interviewed by HCPH investigators. Matched controls were identified during case-patient interviews and were asymptomatic household members who also had attended the church festival. Case-patients were matched to controls using m:n matching (i.e., varying number of case-patients and controls in matched sets). Case-patients and controls were interviewed by telephone using an outbreak-specific survey instrument designed to collect demographic, clinical, and food-exposure data. Thirty-eight case-patients agreed to participate in the study and were interviewed; however, only 23 case-patients could be matched to a household control and included in the matched analysis. Among the 31 controls who agreed to participate, 30 provided adequate food-exposure data for analysis. Thirteen matched sets were generated from the 23 case-patients and 30 controls who were included the matched analysis.

Conditional logistic regression was used to calculate the approximate and exact odds ratios for the association between each food item and illness. Use of exact methods was necessary because pulled pork was a nearly perfect predictor of disease and resulted in a zero cell count (i.e., there were no ill persons who did not eat the pulled pork).

The median age of the case-patients was 44 years (range: 11–72 years), and 19 (50%) were males. This was significantly higher than the median age of controls (15 years; range: 5–67 years) (p<0.001); 16 (52%) of the controls were male. Approximately 89% (34) of case-patients reported an onset of illness during June 13–15, for a median incubation period of 2 days (i.e., days from the end of the church festival to the onset of symptoms) (Figure). Four case-patients reported incubation periods of 3 days (two case-patients), 4 days (one), and 5 days (one). The most frequently reported symptoms were diarrhea (37 case-patients [97%]), cramps (26 [68%]), body ache (23 [61%]), fever (22 [58%]), and headache (21 [55%]). Of those case-patients who had diarrhea, four (11%) reported experiencing bloody diarrhea. The median duration of illness was 5 days among the 26 (68%) case-patients who were no longer symptomatic at the time of interview; seven case-patients who reported antibiotic use also had a median duration of illness of 5 days. Fifteen (40%) case-patients reported seeing a health-care provider since illness onset. No hospitalizations or deaths were reported in the outbreak.

Fifty-one food items served at the church festival were evaluated as sources of exposure in the case-control study. Only pulled pork and coleslaw were identified as statistically significant predictors of the disease associated with the outbreak. Twenty-three (100%) of the case-patients who were included in the matched analysis ate the pulled pork; four (13%) of the 30 controls ate the pulled pork (matched odds ratio = 58.9 [95% confidence interval = 9.4–∞]). The matched odds ratio for consuming coleslaw was 26.2 (95% confidence interval = 3.2–215.7).

All three *Salmonella* isolates were submitted to the Ohio Department of Health laboratory for molecular genotyping and had matching pulsed-field gel electrophoresis (PFGE) patterns identified as *Salmonella* Typhimurium variant Copenhagen (JPXX01.0003). No additional cases were identified through PulseNet, the national molecular subtyping network for foodborne disease surveillance. No food or environmental samples were available for testing.

To assess environmental factors that might have contributed to the outbreak, the pulled pork vendor was interviewed and revealed that the pork was prepared in a private home. The vendor reported that the pulled pork was cooked to an internal temperature of approximately 180°F (82°C), subsequently cooled in pans in a residential-style (i.e., noncommercial) refrigerator, and then reheated at the church festival. The refrigerator internal temperature was said to have been below 41°F (5°C) during cooling, but the vendors were unable to report the time it took for the cooked product to reach a uniform temperature of ≤41°F (≤5°C). The time and temperature parameters of the reheating process also were unknown. After the interviews with the implicated food vendor and discussions with the festival coordinator, it was determined that this vendor’s operation would have been subject to the food service licensing requirements specified by the Ohio Revised Code. Although the vendor was operating at the church festival, the management of the vendor’s proceeds precluded an exemption under Ohio Revised Code. The vendor and festival coordinator were informed of the relevant public health regulations and the associated food safety practices. HCPH used this experience to initiate the development of new outreach and education materials designed specifically to address food safety regulations and concerns related to events and venues, such as church festivals, that are generally exempt from food service licensure and inspection in the state of Ohio.

## Reported by

*Alonzo T. Folger, PhD, Craig S. Davidson, MS, Luke K. Jacobs, MPH, Pat Allingham, MS, Greg E. Kesterman, Hamilton County Public Health, Cincinnati, Ohio.* ***Corresponding contributor:*** *Craig S. Davidson, craig.davidson@hamilton-co.org, 513-946-7617.*

## Editorial Note

Salmonellosis is a common cause of foodborne illness and has contributed to local and national level outbreaks in various settings and environments (2–4). Common risk factors include exposure to contaminated food or water (5). Prevention measures include proper food handling, hand hygiene, and cooking procedures. A breakdown in procedure can increase the risk for foodborne outbreaks during large events.

According to a 2006 study by the U.S. Department of Agriculture’s National Animal Health Monitoring System, the *Salmonella* serotype associated with the outbreak represented 22.6% of all *Salmonella* isolated from swine (6). *Salmonella* Typhimurium variant Copenhagen was the second most common *Salmonella* serotype identified by the national study, although the prevalence was low (6). Because swine are known reservoirs for *Salmonella* Typhimurium, pork products, especially those processed and prepared in unregulated environments, are potential vehicles of disease transmission.

CDC’s Foodborne Outbreak Online Database (FOOD) lists four *Salmonella* outbreaks in three states associated with food consumed at a church, temple, or religious location in 2011 (the most recent year for which FOOD data are available online).* A recent outbreak of salmonellosis after a church barbeque in North Carolina resulted in nine reported, laboratory-confirmed cases with five hospitalizations 10 days later.† Dissemination of risk messages and targeted food-safety education should be a focus for local health departments in and outside of Ohio.

The results of the investigation underscore the risk associated with food service at large-scale events and the importance of rapid investigation to determine the cause of foodborne outbreaks in these environments. Large-scale gatherings provide an opportunity for point-source exposure to foodborne pathogens. Although prevention measures are effective, a breakdown in food handling procedures, such as improperly cooked or stored meat or cross-contamination, can lead to pathogen exposure. Complicating the issue is that food service operations on church grounds are commonly exempt from regulation and licensing in Ohio, excusing these entities from conventional public health inspections conducted as prevention measures (1). The absence of these preventive measures might increase the risk for improper food preparation and handling, increasing the likelihood of foodborne outbreaks. The experience of this outbreak investigation revealed that environments without public health regulation, such as church festivals, might place populations at risk for foodborne illness and might benefit from food safety education of festival organizers and food vendors.

What is already known on this topic?

Festivals and fairs have been implicated in foodborne outbreaks and might remain environments that place persons at risk for foodborne illness. In Ohio, church festivals generally are exempt from food service licensure and inspection.

What is added by this report?

An outbreak of salmonellosis in southwest Ohio in June of 2010 was associated with consumption of pulled pork prepared in a private home and sold to attendees of a church festival. *Salmonella* isolates available from three of 38 reported cases had matching pulsed-field gel electrophoresis patterns identifying the outbreak pathogen as *Salmonella* Typhimurium variant Copenhagen.

What are the implications for public health practice?

Food service operations at large-scale events, including church festivals and fairs, might place populations at risk for foodborne illness. In particular, environments without public health regulation might benefit from education of event organizers and food vendors regarding food safety practices.

## Figures and Tables

**FIGURE:**
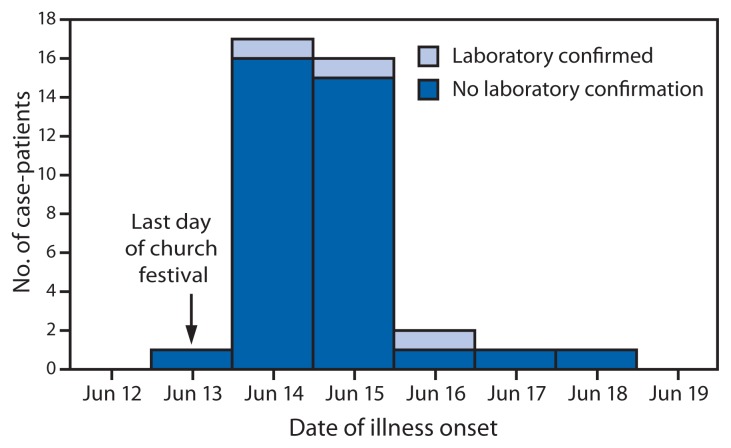
Number of case-patients (N = 38) participating in a matched case-control study after a salmonellosis outbreak associated with a church festival, by date of illness onset and laboratory confirmation status of *Salmonella* infection — Ohio, June 2010

## Footnotes

* Additional information available at http://wwwn.cdc.gov/foodborneoutbreaks/default.aspx.

† Additional information available at http://www.ncdhhs.gov/pressrel/2013/2013-09-17_clev_co_salmonella_outbreak.htm.
